# Supplementary material for: Effect of Dietary Supplementation of Lactiplantibacillus plantarum N-1 and Its Synergies with Oligomeric Isomaltose on the Growth Performance and Meat Quality in Hu Sheep
Source: Foods. 2023 Apr 29;12(9):1858. doi: 10.3390/foods12091858 (PMC10178320; doi:10.3390/foods12091858)
Supplement: Supplementary file 1 [file foods-12-01858-s001.zip › foods-2332503-supplementary.pdf]

Supplementary Table S1 Primer pairs designed for target genes.

Gene	Primer sequences (5′–3′)	GenBank accession number	Annealing temperature (°C)
PPAR-γ	GCATTTCTGCTCCGCACTAC	NM_001100921.1	56.94
	ATACAGGCTCCACTTTGATTGC		55.21
FAS	GGAAGGCGGGACTATATGGC	XM_004013447.1	58.39
	CATGCTGTAGCCTACGAGGG		58.19
HSL	ACAGCAGCGACACAACAGAC	NM_001128154.1	58.45
	CAGATTCATCCTCAGACCCAAG		55.11
SCD	AGTACCGCTGGCACATCAAC	NM_001009254.1	58.17
	AAGACGGCAGCCTTGGATAC		57.76
MAPK	CAGTACTACGATCCAAGCGA	XM_012098101.1	53.82
	GCTGGAATCTAGCAGTCTCT		53.69
FABP4	AAACTGGGATGGGAAATCAACC	NM_001114667.1	55.27
	TGCTCTCTCGTAAACTCTGGTAGC		58.31
LPL	CCAGCAGCATTATCCAGTGTC	NM_001009394.1	56.18
	CCCAAGAGATGCACATTACCC		55.99
PGC-1α	GCGCCGTGTGATTTACGTT	XM_015096414.3	56.49
	AAAACTTCAAAGCGGTCTCTCAA		54.30
ACLY	ATGTCCTGTTCCACCACGAG	XM_012185121.2	57.37
	TTCTTGATGTCCTCGGGATTC		54.21
ACC	GCCGTATGACCCTCGGTG	NM_001009256.1	59.19
	CGGGTATTCCTCCTAGCCTGG		60.01
β-actin	CAGTCGGTTGGATCGAGCAT	NM_001009784.3	57.67
	AGAAGGAGGGTGGCTTTTGG		57.83

PPARγ, Peroxisome proliferator activated receptor gamma; FAS, Fatty acid synthetase; HSL, Hormone sensitive lipase; SCD, Stearoyl-CoA desaturase; MAPK, Mitogen-activated protein kinase; FABP4, Fatty acid binding protein 4; LPL, Lipoprotein lipase; PGC-1α, Peroxisome proliferator-activated receptor-γ coactivator-1α; ACLY, ATP citrate lyase; ACC, Acetyl-CoA carboxylase.
